# A systematic review of the clinical effectiveness of 64-slice or higher computed tomography angiography as an alternative to invasive coronary angiography in the investigation of suspected coronary artery disease

**DOI:** 10.1186/1471-2261-11-32

**Published:** 2011-06-16

**Authors:** Daniel C Paech, Adèle R Weston

**Affiliations:** 1 Health Technology Analysts Pty Ltd, 135 Rowntree St, Balmain NSW 2041, Australia

## Abstract

**Background:**

This systematic review summarized recent evidence pertaining to the clinical effectiveness of 64-slice or higher computed tomography angiography (CTA) in patients with suspected coronary artery disease (CAD). If CTA proves to be a successful diagnostic performance measure, it could prevent the use of invasive diagnostic procedures in some patients. This would provide multiple health and cost benefits, particularly for under resourced areas where invasive coronary angiography is not always available.

**Methods:**

A systematic method of literature searching and selection was employed with searches limited to December 2006 to March 2009. Included studies were quality assessed using National Health and Medical Research Council (NHMRC) diagnostic levels of evidence and a modified Quality Assessment of Diagnostic Accuracy Studies (QUADAS) tool. Individual and pooled diagnostic performance measures were calculated using standard meta-analytic techniques at the patient, vessel and segment level. A positive result was defined as greater than or equal to 50% stenosis.

**Results:**

Twenty-eight studies were included in the systematic review examining 3,674 patients. The primary meta-analysis at the patient-level indicated a sensitivity of 98.2% and specificity of 81.6%. The median (range) positive predictive value (PPV) was 90.5% (76%-100%) and negative predictive value (NPV) 99.0% (83%-100%). In all vessels, the pooled sensitivity was 94.9%, specificity 89.5%, and median (range) PPV 75.0% (53%-95%) and NPV 99.0% (93%-100%). At the individual artery level, overall diagnostic accuracy appeared to be slightly higher in the left main coronary artery and slightly lower in the left anterior descending and circumflex artery. In all segments, the sensitivity was 91.3%, specificity 94.0% and median (range) PPV 69.0% (44%-86%) and NPV 99.0% (98%-100%).

**Conclusions:**

The high sensitivity indicates that CTA can effectively identify the majority of patients with significant coronary artery stenosis. The high NPV at the patient, vessel and segment level establishes CTA as an effective non-invasive alternative to invasive coronary angiography (ICA) for the exclusion of stenosis.

## Background

Coronary artery disease (CAD) is the leading cause of death and disability in the U.S. and other Western countries [1]. Detecting and assessing the extent of CAD has become increasingly important so that early intervention can be applied to decrease morbidity and mortality. When patients present with suspected CAD, a resting electrocardiogram (ECG) is normally performed in the first instance. If this is normal or equivocal, an exercise stress test will usually follow. If this remains inconclusive, invasive coronary angiography (ICA) is often performed. ICA allows *ad hoc *performance of coronary interventions such as percutaneous coronary intervention (PCI), however only one third of all ICAs are performed in conjunction with a revascularisation procedure, whilst the rest are performed only for diagnostic purposes [2].

Due partly to its high cost, but also because of the associated mortality and morbidity, it has been suggested that ICA is not ideal as a widespread diagnostic procedure thus prompting interest in the development of non-invasive coronary imaging [3]. The most serious complications of ICA are death (0.1-0.2%), non-fatal myocardial infarction (MI) (0.1%) and cerebrovascular accidents (0.1%) [4-6]. Other complications include arrhythmias, vasovagal reactions, infections and allergic dye reactions. Exercise ECG is widely used for non-invasive detection of CAD owing to its ready availability and relatively low cost [7]. Other non-invasive techniques such as computed tomography angiography (CTA) have been developed to assist in the risk assessment process. At present, CTA is mainly used for detecting or excluding significant coronary stenosis (≥50% diameter reduction) in coronary arteries. Recently, there has been considerable enhancement in temporal and spatial resolution, which has been reported to result in clinical benefit in terms of improved diagnostic accuracy [8]. The newer multi-detector machines can produce more images in less time, thereby increasing throughput and theoretically decreasing the cost per patient.

Despite this, owing mainly to its high temporal and spatial resolution, ICA remains the diagnostic criterion standard for clinical evaluation of known or suspected CAD. It is conducted both to assist with determining the extent of CAD and to help guide cardiac interventions to treat these disorders. Although CTA is less invasive, carries a lower risk of serious complications, and is cheaper than ICA, concerns about its accuracy in excluding patients without significant stenosis has prevented it becoming a diagnostic test of choice for clinicians [9].

This systematic review aimed to summarise recent evidence pertaining to the clinical effectiveness of 64-slice CTA in patients with suspected CAD. While true that it is based on a previous systematic review, this study provides the most recent diagnostic performance information from studies of 64-slice CT angiography in patients with suspected CAD. It is important to note there has been a significant volume of new literature published on this topic in recent years. If CTA proves to be a successful diagnostic performance measure, it could prevent the application of invasive diagnostic procedures in some patients. It may be of particular value in settings where patients do not have ready access to ICA.

## Methods

### Literature search

This systematic review, commissioned by the New Zealand Ministry of Health, was based on a previous health technology assessment (HTA) of the clinical effectiveness and cost-effectiveness of 64-slice or higher CTA as an alternative to ICA in the investigation of CAD [10]. The HTA report had a search date end of December 2006. For this review, searches were limited to English language material published from between December 2006 and March 2009. The primary computerized search was conducted by cross-searching EMBASE, Medline, the Cochrane library and HTA databases. Individual search strategies for each electronic database, using relevant subject headings, were undertaken based on the literature search by Mowatt *et al *[10]. These included the following keywords: coronary artery disease, myocardial ischemia, ischemic heart disease, myocardial infarction, chest pain, angina, stenosis, computed tomography, computer assisted tomography, computed tomographic angiography, invasive coronary angiography and coronary angiogram.

### Study eligibility

Titles and abstracts of identified studies were screened for possible inclusion or exclusion before retrieving full text versions of the publications. Included studies were those that compared the diagnostic accuracy of CTA to ICA in patients with suspected CAD. As opposed to the broader review by Mowatt *et al *[10], this review did not include prognostic studies, technical studies (e.g. image quality), assessment studies, or post-revascularisation studies. Citations were excluded if they were reported as a conference abstract, not a diagnostic performance study; they included the wrong intervention (i.e. not 64-slice or higher CTA); they did not report diagnostic performance results relating to the identified outcome of interest (≥50% stenosis); or if they had fewer than 50 study participants receiving both CTA and the reference standard. Double-checking of the eligibility of studies by a second reviewer was not undertaken.

Part of the criteria for determining study eligibility was that articles reported either the absolute number of true positives, false positives, false negatives and true negatives, or sensitivity and specificity. Due to the nature of CTA, this could have been presented at the patient, vessel or segment level. Some papers reported diagnostic performance results for individual coronary arteries including the right coronary artery, left coronary artery, left anterior descending and circumflex artery. Most reported the results for all segments with some papers breaking this down further to proximal and distal segments, or side branches.

The level of analysis has implications for clinical practice. Patient-level results are the most important from a patient management perspective because if a significant stenosis is successfully detected then that patient will be referred for an ICA. If results suggest no significant stenosis, then theoretically the patient would not require an ICA and would continue with conservative management. At the vessel-level, if a significant stenosis is detected in a certain artery (i.e. left anterior descending artery), this will better inform the clinician who is to perform the forthcoming intervention. The segment-level analysis is valuable to gauge the diagnostic precision of the technology and can also provide useful information for a re-vascularisation procedure, if it is required.

### Appraisal of included studies

Each of the included studies was reviewed and assigned a level of evidence in accordance with the National Health and Medical Research Council (NHMRC) of Australia diagnostic levels of evidence [11]. In addition, in accordance with the review by Mowatt and colleagues [10], individual study quality for this update was assessed using a modified version of the Quality Assessment of Diagnostic Studies (QUADAS) tool. Quality criteria were tabulated in the data extraction form, rather than used to formulate a numeric score.

### Equivocal test results

The classification of equivocal test results is important in diagnostic intervention studies as it influences the sensitivity, specificity, PPV, NPV and overall diagnostic accuracy of the test. By definition it is the equivocal tests that are most likely to be misclassified. There was considerable variation in how equivocal results were handled in the included studies. Some studies in the current review treated equivocal results as test positive (where the true disease status was known), some as false positive, and some excluded equivocal tests from the analysis completely. Where the true disease status is presented for equivocal test results but not included in the diagnostic performance measures, the reviewer has re-calculated these measures. Excluding equivocal results is the least preferred methodology as it can bias results in favour of the test. In addition, in clinical practice, patients with either positive CTA results or non-evaluable test results will most probably undergo ICA [12].

### Data extraction and data synthesis

In the included studies, a positive CTA or ICA test was defined as ≥50% stenosis. Data were extracted by one reviewer onto specifically designed data extraction forms. The sensitivity and specificity of CTA at the patient-level were meta-analysed using Review Manager Version 5.0 and Metadisc Version 1.4. Pooled estimates were provided together with 95% confidence intervals of the estimate. The pooled estimates correspond to weighted averages in which the weight of each study is its sample size. Pooled confidence intervals are calculated using an F-distribution. In addition, an assessment of diagnostic threshold variation among studies was undertaken using a summary receiver operating characteristic (SROC) curve.

Pooled sensitivity and specificity and median (range) PPV, NPV and overall diagnostic accuracy results were calculated for: all vessels, individual coronary arteries (right coronary; left coronary; left anterior descending; and circumflex arteries) as well as all segments, using the equivocal test result selection methodology outlined above. The analysis of vessels did not include any sub-group analyses that limited results by vessel size. For patient-level analysis, forest plots were also included.

The base-case meta-analysis presented the diagnostic performance results of CTA at the patient-level but omitted studies that excluded equivocal test results. A second (alternative) meta-analysis included all studies that presented patient-level results with treatment of equivocal test results preferentially included as follows. The first preference was results (either as reported by authors or re-calculated by the reviewer) with equivocal CTA test results included and treated as test positive, with disease status as determined by ICA correctly assigned. The second preference was for results presented with equivocal test results treated as false positives (i.e. intent-to-diagnose principal). In this case the ICA result for equivocal segments was unknown and therefore could not be re-calculated. This convention assumes that the test was classified positive and the disease state negative. The third preference was for results presented with equivocal tests excluded.

## Results

### Literature search

There were 1,438 non-duplicate studies identified by the search strategy. Ninety nine full text articles were eligible for retrieval after excluding studies based on their title or abstract. Of the full papers retrieved, 71 did not fulfil the eligibility criteria and were excluded. Therefore, 28 articles examining 3,674 patients were included and fully appraised in this systematic review.

### Characteristics of the included studies

The characteristics of the included studies are summarised in **Table **1. Of the 28 included studies, nine were assessed as Level II diagnostic evidence, 15 were categorised as Level III-1 evidence, and four as Level III-2 evidence [8,12-38]. The majority were diagnostic intervention studies in which patients presenting with suspected CAD were prospectively analysed with 64-slice CTA to determine whether or not they had significant stenosis of coronary arteries. In order to validate the CTA results, all the included studies required patients to undergo a conventional ICA, which in some cases was performed prior to the 64-slice CTA. In all studies, however, the ICA was performed independently of the CTA, and with the exception of two studies, it was clear that the results of the index test had been interpreted by a reviewer blinded to the results of the reference standard.

**Table 1:** Summary of included study characteristics

Author (Year)	Type of scanner	Participants analysed (n)	Mean age (years)	Gender (M/F)	Prevalence of CAD (%)	Mean heart rate (± SD) (bpm)	Level of analysis presented in publication		
	
							Patient	Vessel	Segment
**Level II diagnostic studies**									

Brodoefel *et al *(2008) [8]	Somatom Sensation 64, Siemens, Germany	102	62.0	82/20	62.7	68.2 ± 13.3	No	No	Yes

Cademartiri *et al *(2008a) [13]	Somatom Sensation 64, Siemens, Germany	170	57.5 ^a^	124/46	NR	62.7 ± 10.5	No	No	Yes

Ghostine *et al *(2008) [14]	Somatom Sensation 64, Siemens, Germany	93	65.0	61/32	46.0	73.0 ± 14.0	Yes	No	Yes

Husmann *et al *(2008) [15]	Somatom Sensation 64, Siemens, Germany	88	64.3	48/40	48.9	63.0 ± 9.2	Yes	Yes	Yes

Leber *et al *(2007) [16]	Somatom Sensation 64, Siemens, Germany	90	58.0	57/33	47.7	73.0 ± NR	Yes	No	Yes

Leschka *et al *(2008a) [17]	Somatom Sensation 64, Siemens, Germany	74	61.7	50/24	47.3	67.7 ± 13.3	Yes	Yes	Yes

Leschka *et al *(2008b) [18]	Somatom Sensation 64, Siemens, Germany	114	62.2	73/41	62.3	68.0 ± 13.0	Yes	No	Yes

Rixe *et al *(2009) [19]	Somatom Sensation 64, Siemens, Germany	76	65.5	47/29	52.6	68.0 ± 9.0	Yes	Yes	Yes

Shabestari *et al *(2007) [20]	Somatom Sensation 64, Siemens, Germany	138	63.0	103/35	78.3	65.0 ± NR ^b^	Yes	Yes	Yes

**Level III-1 diagnostic studies**									

Achenbach *et al *(2008) [21]	Somatom Sensation 64, Siemens Germany	200	63.0	114/86	44.5	76 ± 13	Yes	Yes	Yes

Budoff *et al *(2008) [22]	Lightspeed VCT Scanner, GE Healthcare	230	57.0	136/94	24.8	60 ± 12	Yes	Yes	No

Cademartiri *et al *(2007) [23]	Somatom Sensation 64, Siemens, Germany	72	53.9	38/34	28.0	70.0 ± 9.9	Yes	Yes	Yes

Hausleiter *et al *(2007) [24]	Somatom Sensation 64 Cardiac, Siemens, Germany	243	62.0	158 ^c/^85	42.0	56.6 ± 6.5	Yes	Yes	Yes

Herzog *et al *(2007) [25]	Somatom Sensation 64, Siemens, Germany	55	67.0	29/26	34.5	64.0 ± NR	Yes	Yes	Yes

Meijboom *et al *(2007a) [26]	Somatom Sensation 64, Siemens, Germany	104	58.7 ^a^	75/29	85.0	66.0 ± 9.0	Yes	Yes	Yes

Meijboom *et al *(2007b) [27]	Somatom Sensation 64; Siemens, Germany	402	59.2 ^a^	279/123	62.9	59.5 ^a ^± NR	Yes	Yes	Yes

Meijboom *et al *(2007c) [28]	Somatom Sensation 64, Siemens, Germany	254	59.0 ^a^	171/83	49.6	59.3 ^a ^± NR	Yes	Yes	Yes

Meijboom *et al *(2008) [12]	Somatom Sensation 64, Siemens, Germany; Brilliance 64, Philips, The Netherlands; Toshiba Multi-Slice Aquilion 64, Toshiba, Japan	360	60.0	245/115	68.0	59 ± 9	Yes	Yes	Yes

Oncel *et al *(2007) [29]	Somatom Sensation 64, Siemens, Germany	80	56.0	61/19	77.5	58.0 ± 10.0	Yes	No	Yes

Piers *et al *(2008) [30]	Somatom Sensation 64, Siemens, Germany	60	64.0	51/9	63.3	63.0 ± 12.0	Yes	Yes	Yes

Scheffel *et al *(2008) [31]	Somatom Sensation 64, Siemens, Germany	120	68.2	71/49	55.0	59.0 ± 6.0	Yes	Yes	Yes

Schlosser *et al *(2007) [32]	Somatom Sensation 64, Siemens, Germany	61	62.4	41/20	NR	57.0 ± 4.0	No	Yes	Yes

Sheth *et al *(2008) [33]	Toshiba Aquilion 64-detector scanner, Toshiba, Japan	80	56.0	43/37	39.5	NR	Yes	Yes	Yes

Weustink *et al *(2007) [34]	DSCT Somatom Sensation 64, Siemens, Germany.	100	61.0	79/21	77.0	68.0 ± 11.0	Yes	Yes	Yes

**Level III-2 diagnostic studies**									

Cademartiri *et al *(2008b) [35]	Somatom Sensation 64 Cardiac; Siemens, Germany	134	63.4	98/36	62.7	57.5 ^a ^± NR	Yes	Yes	Yes

Han *et al *(2008) [36]	64-slice VCT; GE Healthcare	53	59.6	228 ^d^/175	81.1	NR	No	No	Yes

Pugliese *et al *(2008) [37]	Somatom Sensation 64, Siemens, Germany	51	59.0	39/12	74.5	58.0 ± 7.0	Yes	No	Yes

Yoshida *et al *(2009) [38]	Somatom Sensation 64, Siemens, Germany	70	64.0	50/20	NR	65.0 ± 11.0	No	No	Yes

Bpm, beats-per-minute; CAD, coronary artery disease; F, female; M, male; NR, not reported.

Levels of evidence were defined according to the NHMRC diagnostic levels of evidence (11)

^a ^Calculated *post hoc *using a crude weighted average of the two reported sub-groups

^b ^Median heart rate

^c ^There was a discrepancy between the number stated in Table 1 of the publication and the number stated in the text (228 and 226, respectively).

^d ^For all enrolled patients in study

Of the included studies, nine recruited patients consecutively whereas 17 either recruited patients non-consecutively or did not report the method of recruitment and were consequently assumed to have recruited non-consecutively. Two included studies were retrospective analyses. In accordance with eligibility criteria determined *a priori*, each included study examined more than 50 patients, with the number of participants analysed ranging from 51 to 402. Consistent with the gender and demographic profile for CAD, more males than females were examined in the included studies with mean age ranging from 53.9 to 68.2 years.

The eligibility criteria of the included studies were similar throughout. Although most studies did not explicitly report inclusion criteria, the study populations included patients with suspected CAD because of a range of symptoms (e.g. angina), usually scheduled for ICA. Those who had undergone a previous PCI such as stenting or CABG were excluded. Patients who had a contraindication to CTA such as a known allergy to iodinated contrast agent were also excluded. Other common exclusion criteria included atrial fibrillation, impaired renal function and inability to follow the breath hold command required to complete the test. There were no exclusions of patients based on significant calcification of the arteries. The majority of patients were given beta-blockers prior to scanning in order to reduce their heart rate.

The prevalence of CAD in the included study cohorts ranged from 24.8%-85.0%. The lowest prevalence (24.8%) came from the study by Budoff *et al *[22] who examined patients presenting with typical or atypical chest pain, who were being referred for ICA. The highest prevalence came from a study by Meijboom and colleagues [26] who investigated high and low risk non-ST segment elevation in acute coronary syndrome patients with a positive or inconclusive exercise ECG test or high suspicion for CAD. Patients presenting with ST-segment elevation MI were excluded. Five other included studies had a prevalence of CAD above 70.0%. All five included patients presenting with typical or atypical chest pain but two studies also included patients with unstable angina. Although all 28 included studies examined patients suspected of having CAD, the variation in prevalence reflects the level of suspicion of significant stenosis.

### Diagnostic accuracy of CTA

**Figure 1:**
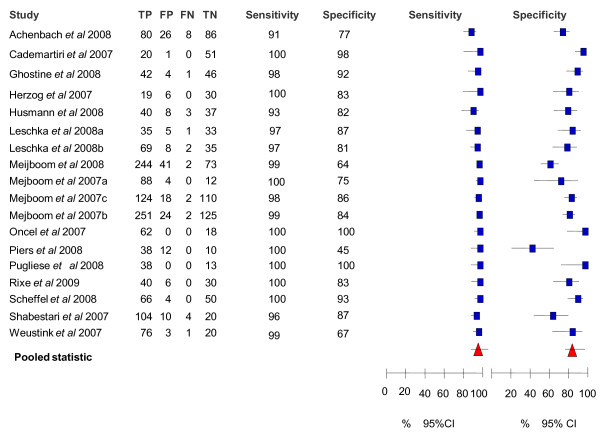
**Sensitivity and specificity for the base case meta-analysis at the patient level**. CI, confidence interval; FN, false negative; FP, false positive; TN, true negative; TP, true positive.

**Figure 2:**
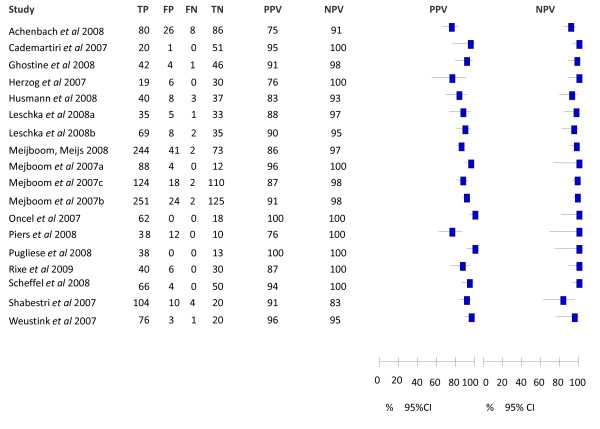
**PPV and NPV for the base case met-analysis at the patient level**. CI, confidence interval; FN, false negative; FP, false positive; TN, true negative; TP, true positive.

Forest plots with the associated sensitivity, specificity, PPV and NPV of CTA for detecting significant stenosis for the patient-level base case meta-analysis are shown in **Figure **1 and **Figure **2. The pooled value (95% CI) for sensitivity and specificity and median (range) for PPV, NPV and overall diagnostic accuracy for all levels of analysis (i.e. patient, vessel and segment level) are presented in **Table **2.

**Table 2:** CTA diagnostic performance measures

Analysis level	No. of included studies ^a^	Sensitivity % (95% CI)	Specificity % (95% CI)	PPV Median (range)	NPV Median (range)	Diagnostic accuracy Median (range)
Patient: base case analysis	18	98.2 (97.4-98.8)	81.6 (79.0-84.0)	90.5 (76-100)	99.0 (83-100)	92.0 (80-100)

Patient: alternative analysis	22	98.0 (97.2-98.6)	83.2 (81.1-85.2)	89.0 (63-100)	98.0 (83-100)	92.0 (80-100)

Vessels: all	17	94.9 (93.9-95.8)	89.5 (88.8-90.2)	75.0 (53-95)	99.0 (93-100)	91.5 (74-98)

RCA	8	94.8 (92.0-96.9)	91.0 (89.0-92.7)	84.0 (73-94)	98.5 (95-100)	94.5 (84-99)

LM	8	95.7 (85.2-99.5)	97.1 (95.7-98.1)	89.0 (24-100)	100.0 (98-100)	99.0 (91-100)

LAD	7	97.4 (95.3-98.8)	84.5 (82.1-86.7)	78.0 (57-95)	99.0 (95-100)	93.0 (72-99)

CX	8	94.1 (90.7-96.6)	89.6 (87.7-91.3)	78.5 (52-90)	99.5 (95-100)	94.0 (75-99)

Segments: all	17	91.3 (90.2-92.2)	94.0 (93.7-94.2)	69.0 (44-86)	99.0 (98-100)	95.5 (90-99)

CI, confidence interval; CX, circumflex; LAD, left anterior descending; LM, left main; NPV, negative predictive value; PPV, positive predictive value; RCA, right coronary artery

^a ^Maximum number of included studies from which data were drawn to calculate diagnostic performance measures

At the patient-level, there were 18 studies included in the base case meta-analysis and 22 studies included in the alternative meta-analysis. Omitting studies that excluded equivocal test results (i.e. the base case), sensitivity in the included studies ranged from 90.9% to 100.0%, with a pooled sensitivity of 98.2% (97.4%-98.8%). Specificity ranged from 45.5% to 100.0%, with a pooled specificity of 81.6% (79.0%-84.0%). The median PPV for the included studies was 90.5% (75.5%-100.0%), with the median NPV 99.0% (83.3%-100.0%). There was little difference between the results of the base case and the alternative analysis.

At the overall vessel-level, omitting studies that excluded equivocal test results, sensitivity ranged from 87.3% to 100.0%, specificity 68.0% to 97.1%, PPV 53.4% to 95.0%, NPV 92.7% to 100.0%, and overall diagnostic accuracy from 73.8% to 98.0%. The results for overall vessel-level analysis showed that the diagnostic performance is similar to that of the patient-level analysis with a high pooled sensitivity and median NPV (94.9% and 99.0%, respectively). The sensitivity for the right coronary artery (RCA) ranged from 89.5% to 100.0%, specificity 72.2% to 99.5%, PPV 72.5% to 94.4%, NPV 95.1% to 100.0% and overall diagnostic accuracy 83.7% to 99.0%. For the left main (LM) artery, the sensitivity ranged from 83.3% to 100.0%, specificity 91.1% to 100.0%, PPV 23.8% to 100.0%, NPV 97.6% to 100.0%, and overall diagnostic accuracy 90.9% to 100.0%. For the left anterior descending (LAD) artery, the sensitivity ranged from 93.8% to 100.0%, specificity 55.8% to 92.7%, PPV 57.1% to 95.0%, NPV 95.0% to 100.0% and overall diagnostic accuracy 71.9% to 99.0%. For the circumflex (CX) artery, the sensitivity ranged from 86.6% to 100.0%, specificity 70.3% to 91.5%, PPV 51.5% to 89.7%, NPV 94.7% to 100.0% and overall diagnostic accuracy 75.3% to 99.0%.

Pooled sensitivity remained high for each individual vessel (94.1% to 97.4%). Specificity was slightly higher in the LM artery 97.1% (95.7%-98.1%) and slightly lower in the LAD artery 84.5% (82.1%-86.7%) compared with the other arteries. The median PPV was similar across vessels except for the LM, in which it was slightly higher 89.0% (24.0%-100.0%). The median NPV was consistently high in each vessel (98.5% to 100.0%).

At the segment-level, sensitivity ranged from 80.7% to 100.0%, specificity 90.2% to 99.1%, PPV 43.6% to 86.4%, NPV 97.9% to 100.0% and overall diagnostic accuracy ranged from 90.0% to 99.1%. There were 17 studies reporting overall segment level results. The pooled sensitivity was 91.3% (90.2%-92.2%), specificity 94.0% (93.7%-94.2%), PPV 69.0% (44.0%-86.0%), NPV 99.0% (98.0-100.0%) and overall diagnostic accuracy 95.5% (90.0%-99.0%).

### Diagnostic threshold effect

**Figure 3:**
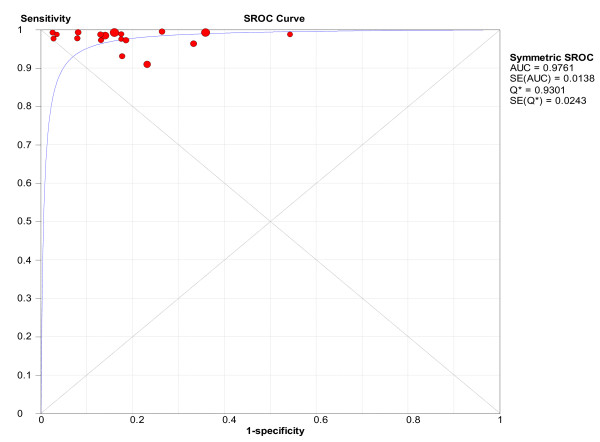
**Summary receiver operator characteristic (SROC) curve for the base case patient level analysis**.

To assess the impact of diagnostic threshold variation between studies a symmetrical SROC curve was fitted (**Figure **3). The area under the curve (± SE) was 0.976 (± 0.014), suggesting that variations in definition of a positive CTA result did not have an influence on the pooled sensitivity and specificity results.

## Discussion

There has been considerable enhancement in temporal and spatial resolution of CTA in recent years, and these improvements appear to have contributed to the fact that CTA now preserves a high rate of evaluable patients. This systematic review summarized, through meta-analysis, recent clinical evidence pertaining to the use of 64-slice CTA in patients with suspected CAD.

The most important results from a patient management perspective are the patient-level results. If this technology is to be used successfully as a triage tool then any patient without significant stenosis would not be referred for an ICA. This may be of particular value when access to ICA is limited. The pooled estimate (95% CI) of sensitivity was high with CTA correctly identifying the majority of patients who had a significant stenosis (98.2%, 97.4%-98.8%). Perhaps more importantly, the median (range) NPV was very high (99.0%, 83-100%). NPV was above 95.0% in 14 of 18 included studies, and above 90.0% in 17 of 18 included studies suggesting that it can reliably exclude patients that test negative as not having significant stenosis. The only exception was the NPV result reported in Shabestri *et al *[20] where the NPV was 83.0%. No reasons were offered by the authors as to why NPV was lower than in previously reported studies. This study had a high prevalence of CAD and subsequently a relatively low number of patients classified as true negative, which may have been why the false negative findings (four in total) significantly influenced the NPV.

The results of the current review were similar to that reported by Mowatt *et al *[10], the systematic review that the current review sought to update. Those authors found that sensitivity ranged from 94% to 100% with a pooled sensitivity of 99% (95% credible interval (CrI) 97.0% to 99.0%). Specificity ranged from 50.0% to 100.0%, with a pooled specificity of 89.0% (95% CrI 83.0% to 94.0%). Across studies the median PPV was 93.0% (range 64.0% to 100.0%), while the median NPV was 100.0% (range 86.0% to 100.0%). Comparisons with this review are limited by the fact that Mowatt and colleagues [10] included patient populations with both suspected and known CAD, rather than just suspected CAD as was the case in the present review. A further six systematic reviews were identified on this topic, all of which had been published during or prior to 2008 [39-44]. Although the reviews varied slightly in terms of their inclusion criteria and analyses, the broad conclusions were consistent with the current systematic review. The reviews suggested high diagnostic accuracy of CTA validates this scanning technique as an alternative to coronary angiography in populations suspected for coronary stenosis.

It has been suggested that CTA may be able to replace other non-invasive diagnostic imaging modalities such as myocardial perfusion scintigraphy (MPS) and stress ECG. A systematic review of single photon emission computed tomography (SPECT) MPS and stress ECG for the diagnosis and management of angina and MI, found sensitivity ranged from 63.0% to 93.0% (median 81.0%) for SPECT and from 42.0% to 92.0% (median 65.0%) for stress ECG. Specificity ranged from 10.0% to 90.0% (median 65.0%) for SPECT and 41.0% to 88.0% (median 67.0%) for stress ECG [7]. The range and pooled estimate of sensitivity and specificity for the base-case meta-analysis was higher in the current review suggesting that CTA may have higher diagnostic accuracy than SPECT MPS and stress ECG in patients with suspected CAD.

The lowest specificity (45.5%) and clear outlier in **Figure **1 was reported by Piers *et al *[30]. The authors attributed this low percentage to the tendency of CTA to overestimate lesion severity (i.e. a large number of false positives). A lower specificity was generally related to difficulties in grading the severity of stenosis, particularly in studies that included all available segments regardless of image quality. The PPV was correlated with the variation in prevalence of CAD in each study cohort. As expected, those studies with a higher prevalence of CAD in the cohort tended to have higher PPVs.

The vessel level results are important because clinicians can identify which arteries contain significant stenosis and match potential re-vascularisation procedures accordingly. It is also important to know if CTA is particularly unreliable in a certain vessel. The pooled results for overall vessel-level analysis showed that the diagnostic performance is similar to that of the patient-level analysis with a high sensitivity 94.9% (93.9-95.8%) and median NPV 99.0% (93.0-100.0%). Sensitivity remained high for each individual vessel. Specificity was slightly higher in the LM artery and slightly lower in the LAD artery, compared with the other arteries. The PPV was similar across vessels except for the LM artery, in which it was slightly higher. The NPV was consistently high in each vessel, suggesting that CTA is no less reliable at excluding significant stenosis in a particular artery.

The segment level results provide the most information on the overall technical accuracy of CTA in detecting significant stenosis in patients with suspected CAD. The sensitivity of the segment level analysis is lower than that of the vessel and patient-level analysis. This may be because there is an increased number of equivocal test results and therefore an increased proportion of incorrectly classified segments (i.e. false negatives). However, specificity is higher at the segment level, probably due to the increased number of true negatives being proportionally higher than the increase in false positives. PPV is significantly lower at the segment level compared with the patient-level because of the increased chance of poor image quality and therefore false positive outcomes. Also, because the conservative approach for clinicians reading CTA results is to treat equivocal tests as false positive. Similar to the other levels of analysis, NPV is high confirming the reliability of 64-slice CTA at excluding those without significant stenosis.

The evidence considered in this review exhibited some methodological limitations. A large variety of studies are included, such as studies dedicated to unstable populations, studies exploring different acquisition modes (prospective triggering) and as such caution should be warranted in interpreting results. Furthermore, the variability in prevalence of CAD in the included studies was a limitation, particularly because of the effect it had on the PPV. Although strict study eligibility and inclusion criteria were applied to try to minimise this, variations in the classification of suspected CAD by the authors of the included studies and subsequent variability in prevalence of disease could not be avoided. Futhermore, the current review does not address the willingness of clinicians to act upon negative CTA results to avoid referral to ICA.

In addition, no studies of 256-slice CTA met the inclusion criteria for the current review. Therefore, conclusions on the usefulness of 256-slice CTA in detecting significant stenosis cannot be made. Due to the abundance of studies conducted using 64-slice CTA since December 2006 and methodological issues with the meta-analyses of non-homogenous patient groups, the patient population investigated was restricted in the current review. As a consequence, the usefulness of CTA in asymptomatic patients or patients with known CAD was not examined.

## Conclusions

Adoption of a new therapeutic strategy should be based on both clinical and cost-effectiveness. This review focused on the clinical effectiveness of CTA and results support previous findings that concluded that the main value of 64-slice CTA is to rule out significant CAD. The high NPV observed at the patient, vessel and segment level establishes CTA as a highly effective non-invasive alternative to ICA for the exclusion of obstructive coronary artery stenosis. It should also be noted, however, that overall diagnostic accuracy varied at the individual artery level, with results being slightly worse for the LAD and CX arteries compared with the RC and LM arteries. It is unlikely that CTA will replace ICA in assessment for revascularisation of patients, particularly as angiography and angioplasty are often performed on the same occasion. However, for those patients who are candidates for standalone diagnosis with ICA, CTA may be a viable alternative. Furthermore, in under serviced and under resourced health areas, where invasive coronary angiography is not always available, CTA appears a viable alternative.

## List of abbreviations

CAD: Coronary artery disease; CrI: Credible interval; CTA: Computed tomography angiography; CX: Circumflex; ECG: Electrocardiogram; HTA: Health technology assessment; ICA: Invasive coronary angiography; MI: Myocardial infarction; NHMRC: National Health and Medical Research Council; NPV: Negative predictive value; PCI: Percutaneous coronary intervention; PPV: Positive predictive value; QUADAS: Quality assessment of diagnostic accuracy studies; RCA: Right coronary artery; LM: Left main; LAD: Left anterior descending

## Competing interests

The authors declare that they have no competing interests.

## Authors' contributions

All authors read and approved the final manuscript. The requirements for authorship stipulated by the International Committee of Medical Journal Editors have been met by all authors. DP contributed significantly to conception and design, contributed to the analysis and interpretation of data and drafted the article. AW contributed substantially to conception and design, revised the manuscript critically for important intellectual content and gave final approval for its publication.

## Pre-publication history

The pre-publication history for this paper can be accessed here:

http://www.biomedcentral.com/1471-2261/11/32/prepub
